# A Modified Delta-Shaped Gastroduodenostomy in Totally Laparoscopic Distal Gastrectomy for Gastric Cancer: A Safe and Feasible Technique

**DOI:** 10.1371/journal.pone.0102736

**Published:** 2014-07-14

**Authors:** Changming Huang, Mi Lin, Qiyue Chen, Jianxian Lin, Chaohui Zheng, Ping Li, Jianwei Xie, Jiabin Wang, Jun Lu

**Affiliations:** Department of Gastric Surgery, Fujian Medical University Union Hospital, Fuzhou, Fujian Province, China; Taipei Medical University, Taiwan

## Abstract

**Background:**

The present study introduced a modified delta-shaped gastroduodenostomy (DSG) technique and assessed the safety, feasibility and clinical results of this procedure in patients undergoing totally laparoscopic distal gastrectomy (TLDG) for gastric cancer (GC).

**Materials and Methods:**

A total of 102 patients with distal GC undergoing TLDG with modified DSG between January 2013 and December 2013 were enrolled. A retrospective study was performed using a prospectively maintained comprehensive database to evaluate the results of the procedure. Univariate and multivariate analyses were performed to estimate the predictive factors for postoperative morbidity.

**Results:**

The mean operation time was 150.6±30.2 min, the mean anastomosis time was 12.2±4.2 min, the mean blood loss was 48.2±33.2 ml, and the mean times to first flatus, fluid diet, soft diet and postoperative hospital stay were 3.8±1.3 days, 5.0±1.0 days, 7.4±2.1 days and 12.0±6.5 days, respectively. Two patients with minor anastomotic leakage after surgery were managed conservatively; no patient experienced any complications around the anastomosis, such as anastomotic stricture or anastomotic hemorrhage. Univariate analysis showed that age, gastric cancer with hemorrhage and cardiovascular disease combined were significant factors that affected postoperative morbidity (P<0.05). Multivariate analysis found that gastric cancer with hemorrhage was the independent risk factor for the postoperative morbidity (P = 0.042). At a median follow-up of 7 months, no patients had died or experienced recurrent or metastatic disease.

**Conclusions:**

The modified DSG was technically safe and feasible, with acceptable surgical outcomes, in patients undergoing TLDG for GC, and this procedure may be promising in these patients.

## Introduction

It has been more than 20 years since the first laparoscopic surgery for gastric cancer (GC). [1] Although reconstruction of the digestive tract is important during the procedure, it is technically difficult and requires a highly skilled surgeon; the Billroth-I (B-I) anastomosis after totally laparoscopic distal gastrectomy (TLDG) is considered especially complex. A method for intracorporeal B-I anastomosis, called delta-shaped gastroduodenostomy (DSG) that uses only endoscopic linear staplers, was first reported in 2002. [2] However, because the higher technical demand is needed and most surgeons still doubt for its safety, this method has only been accepted in some Asian countries, such as Japan and Korea [3]–[5], and it has not been carried out extensively at present. Our institution has performed this method since November 2012. During the implementation process, we reviewed the experiences based on the anatomy and anastomotic characteristics. In order to simplify the operation procedures to obtain a simpler process and reduce the potential risk as far as possible to increase the safety of operation, the modified DSG was therefore proposed with the hope that the method can be accepted and generalized by more surgeons. Here, we introduce this modified DSG and evaluate its safety, feasibility and clinical results in patients undergoing TLDG for GC.

## Materials and Methods

### Patients

Between January 2013 and December 2013, 102 patients with primary distal GC underwent modified DSG along with TLDG in the Department of Gastric Surgery, Fujian Medical University Union Hospital. All operations were performed by the same surgeon who had experience performing more than 2000 cases of laparoscopic gastrectomy and was proficient at laparoscopic surgery. Distal GC was diagnosed by the analysis of endoscopic biopsy specimens. The pretreatment tumor site, depth of invasion, extent of lymph node (LN) metastasis and metastatic disease were evaluated by endoscopy, computed tomography (CT), ultrasonography of the abdomen and/or chest radiography. Patients with distant metastases were excluded.

### Surgical procedures

**Figure 1:**
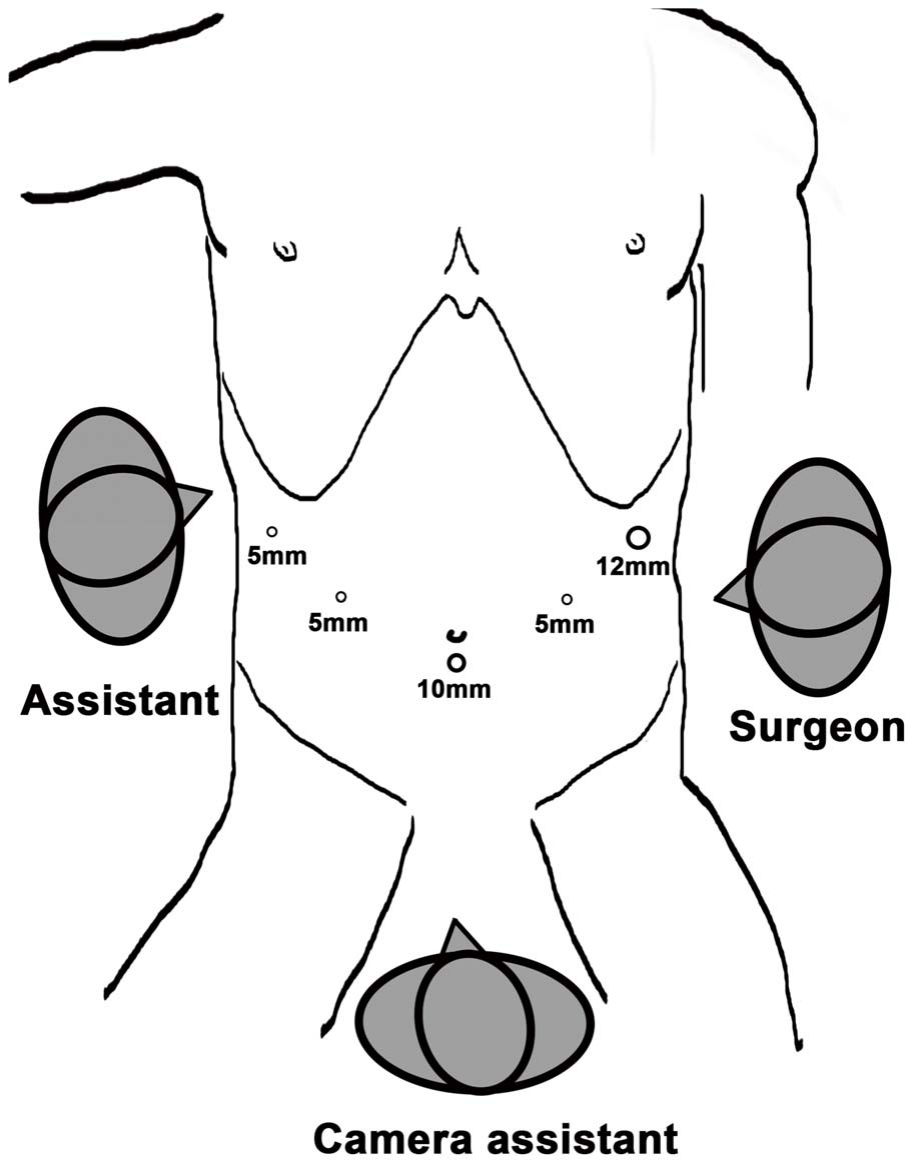
Trocar placements for totally laparoscopic distal gastrectomy.

All patients voluntarily chose laparoscopic surgery and provided written informed consent prior to surgery. The gastroduodenostomy was reconstructed using an endoscopic linear stapler (ECHELON 60; Ethicon Endo-Surgery, Cincinnati, OH, USA). Routine preoperative preparation was performed. Under general anesthesia with endotracheal intubation, the patient was placed in the reverse Trendelenburg position with the legs apart and head elevated approximately 10 to 20 degrees. A 10-mm trocar for the laparoscope was inserted 1 cm below the umbilicus, and a 12-mm trocar was introduced in the left preaxillary line 2 cm below the costal margin as a major hand port; a 5-mm trocar was inserted in the left midclavicular line 2 cm above the umbilicus as an accessory port, and a second 5-mm trocar was placed at the contralateral site. A third 5-mm trocar was inserted in the right preaxillary line 2 cm below the costal margin for exposure. Carbon dioxide pneumoperitoneum with 12–14 mmHg was established. The surgeon stood on the patient’s left and the assistant stood on the patient’s right side. The camera assistant was placed between the patient’s legs. (Fig. 1).

It was routine to first confirm the tumor site and determine if there was T4b GC or metastasis to the liver, abdominal cavity, or pelvic cavity based on laparoscopic exploration. If it was difficult to identify the tumor site in the early tumors during total laparoscopy, the intraoperative gastroscopy could be used for accurate positioning to ensure R0 tumor resection. LN dissection was performed according to the guidelines of the Japanese Gastric Cancer Association. [6] The greater curvature of the stomach was dissected with the preservation of the posterior gastric vessels and 2–3 branches of the short gastric vessels. Then, the duodenum was fully mobilized to 2.5–4 cm distal to the pylorus.

**Figure 2:**
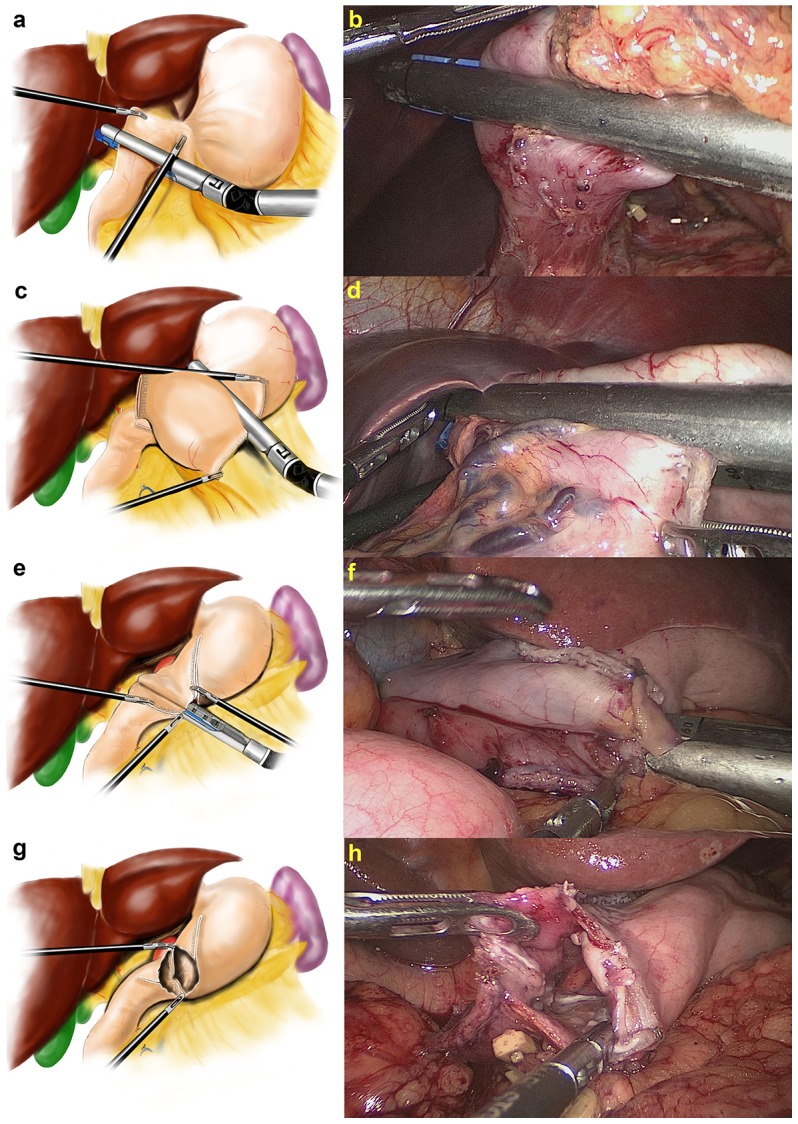
The procedures of modified delta-shaped gastroduodenostomy before closing the common stab incision. **a** Diagram showing that the stapler was positioned across the duodenum vertical to the long axis in the predetermined position and fired to transect the duodenum by rotating 90 degrees from back to front. **b** Intraoperative image showing that the stapler was positioned across the duodenum vertical to the long axis in the predetermined position and fired to transect the duodenum by rotating 90 degrees from back to front. **c** Diagram showing that the stomach was resected by successively transecting from the greater curvature to the lesser curvature with two staplers. **d** Intraoperative image showing that the stomach was resected by successively transecting from the greater curvature to the lesser curvature with two staplers. **e** Diagram showing that the stapler was positioned to join the posterior walls together. **f** Intraoperative image showing that the stapler was positioned to join the posterior walls together. **g** Diagram showing the V-shaped anastomosis on the posterior wall. **h** Intraoperative image showing the V-shaped anastomosis on the posterior wall.

During the reconstruction of the digestive tract, the trocar site, the patient position and the surgeon’s position were all the same as during the LN dissection. The gastric tube should be at 40 cm before the anastomosis. An endoscopic linear stapler was inserted through the left upper major hand port, positioned across the duodenum vertical to the long axis in the predetermined position, and fired to transect the duodenum by rotating 90 degrees from back to front. The stomach was then resected by successively transecting from the greater curvature to the lesser curvature with two staplers. A suitably sized remnant stomach should be produced to ensure not only R0 tumor resection but also appropriate anastomotic tension. After the specimen was placed into a plastic specimen bag intracorporeally, small incisions were made on the greater curvature of the remnant stomach and the posterior side of the duodenum. Due to the greater mobility of the stomach, one limb of the stapler was first inserted into the stomach incision and the other limb was positioned on the duodenum. The cutting edge of the duodenum was rotated 90 degrees counterclockwise. Following approximation of the posterior walls of the gastric remnant and duodenum, with a distance of approximately 2 cm between the predetermined anastomotic line and the gastric cutting edge, the forks of the stapler were closed and fired, creating a V-shaped anastomosis on the posterior wall. Confirmation was made via the common stab incision that there was no bleeding of the anastomosis or no injury to the duodenal mucosa. (Fig. 2).

**Figure 3:**
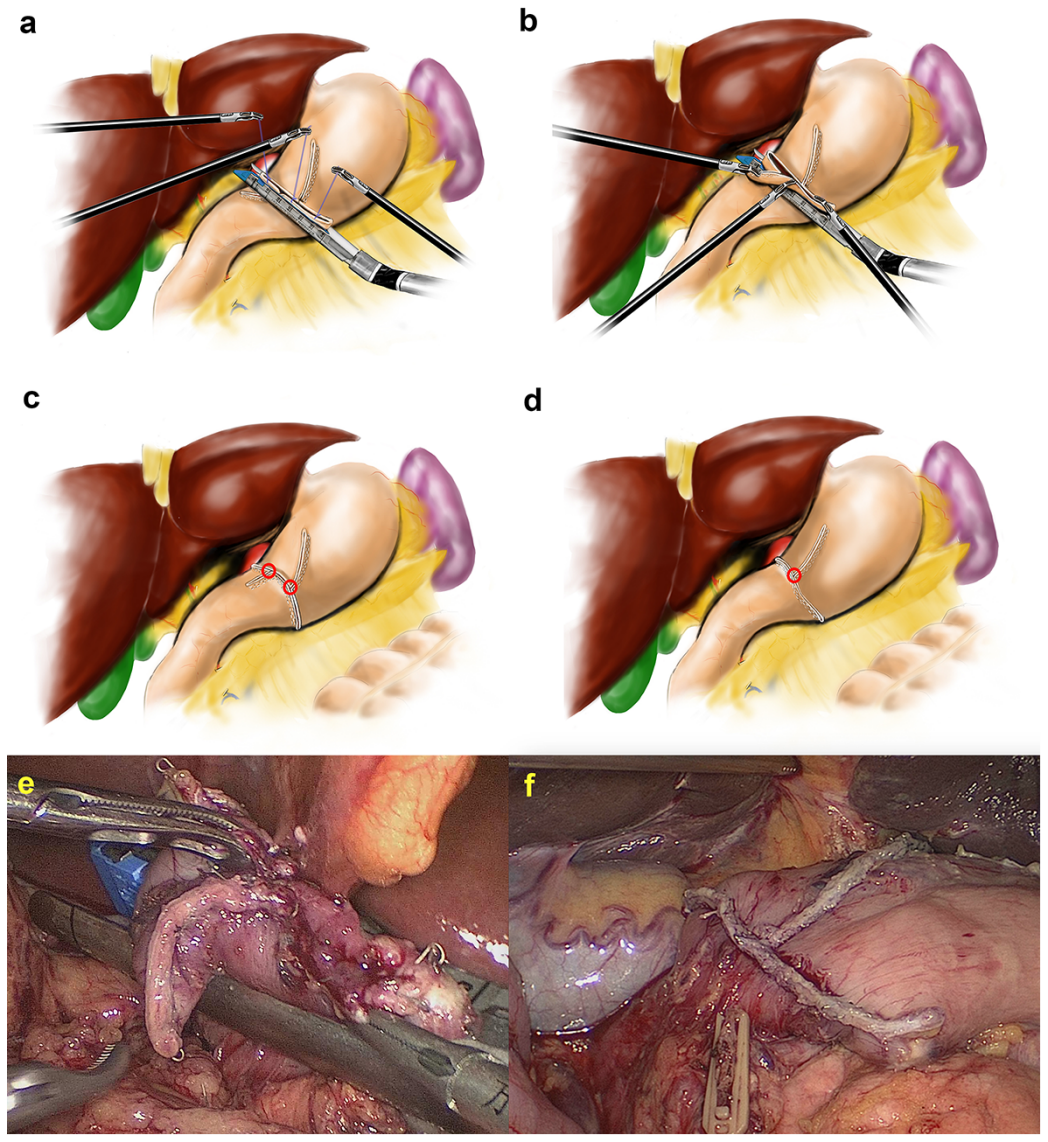
The differences between the conventional delta-shaped gastroduodenostomy (DSG) and the modified DSG. **a** Diagram showing that three sutures were added to each end of the common stab incision and the cutting edges of the stomach and duodenum to obtain an involution and pull in the conventional DSG. **b** Diagram showing the completed involution of the common stab incision using the instruments of the surgeon and assistant with the blind angle of the duodenum being pulled up into the stapler in the modified DSG. **c** Diagram showing the completed conventional DSG with two intersections of the gastroduodenal cutting edge and the common closed edge. **d** Diagram showing the completed modified DSG with only one intersection of the gastric cutting edge and the common closed edge. **e** Intraoperative image showing the completed involution of the common stab incision using the instruments of the surgeon and assistant with the blind angle of the duodenum being pulled up into the stapler in the modified DSG. **f** Intraoperative image showing the completed inverted T-shaped appearance of anastomosis in the modified DSG.

This procedure was different from the conventional DSG during closing the common stab incision of the stomach and the duodenum. Three sutures added to each end of the common stab incision and the cutting edges of the stomach and duodenum to obtain an involution and pull were omitted. Instead, the lower end of the common stab incision was grasped with the surgeon’s left forceps, and the other end was pulled by the assistant’s left forceps to flatten it. At the same time, the surgeon, using his right hand, inserted the endoscopic linear stapler to close around the common stab incision and produce an involution. The other end of the duodenal cutting edge, defined as the blind angle was pulled up into the stapler by the assistant’s right forceps, with the two hands coordinating with each other to obtain a better involution. Therefore, the blind angle of the duodenum was completely resected at the same time when the common stab incision was closed with the stapler. In this way, the two intersections of the gastroduodenal cutting edge and the common closed edge in the conventional DSG were reduced to only one intersection of the gastric cutting edge and the common closed edge. To avoid anastomotic stricture, the direction of the common closed edge should be perpendicular to the cutting edge of the stomach. After the anastomotic tension and quality was checked, a secure suture was added to reinforce the anastomosis if anastomosis oozing occurs. The reconstruction of the intracorporeal digestive tract was accomplished. The anastomosis appeared as an inverted T-shape, which was different from the conventional DSG. (Fig. 3).

### Data collection

**Figure 4:**
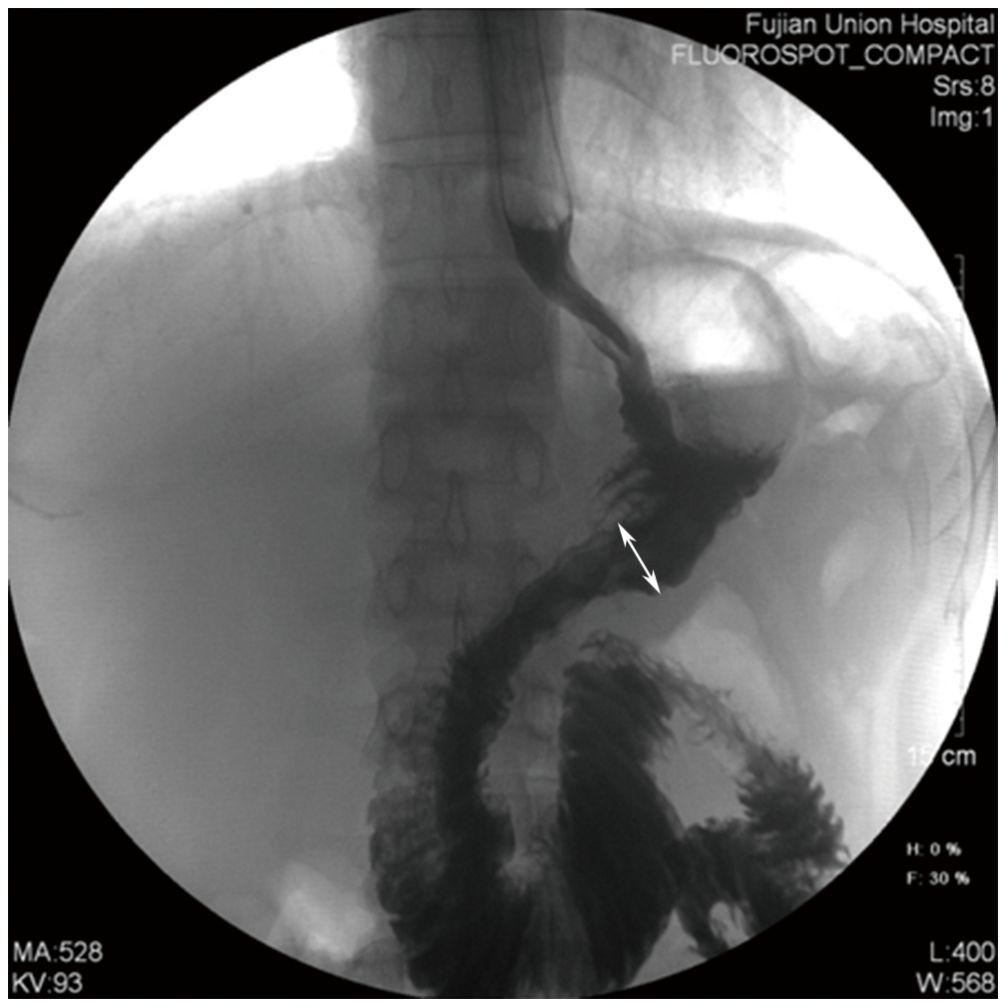
The film of upper gastrointestinal radiography. The film of upper gastrointestinal radiography with diatrizoate meglumine as the contrast medium on postoperative day 7 for one patient underwent the modified delta-shaped gastroduodenostomy. The inner diameter of the anastomosis was measured the length of the white arrow as shown in the figure.

**Figure 5:**
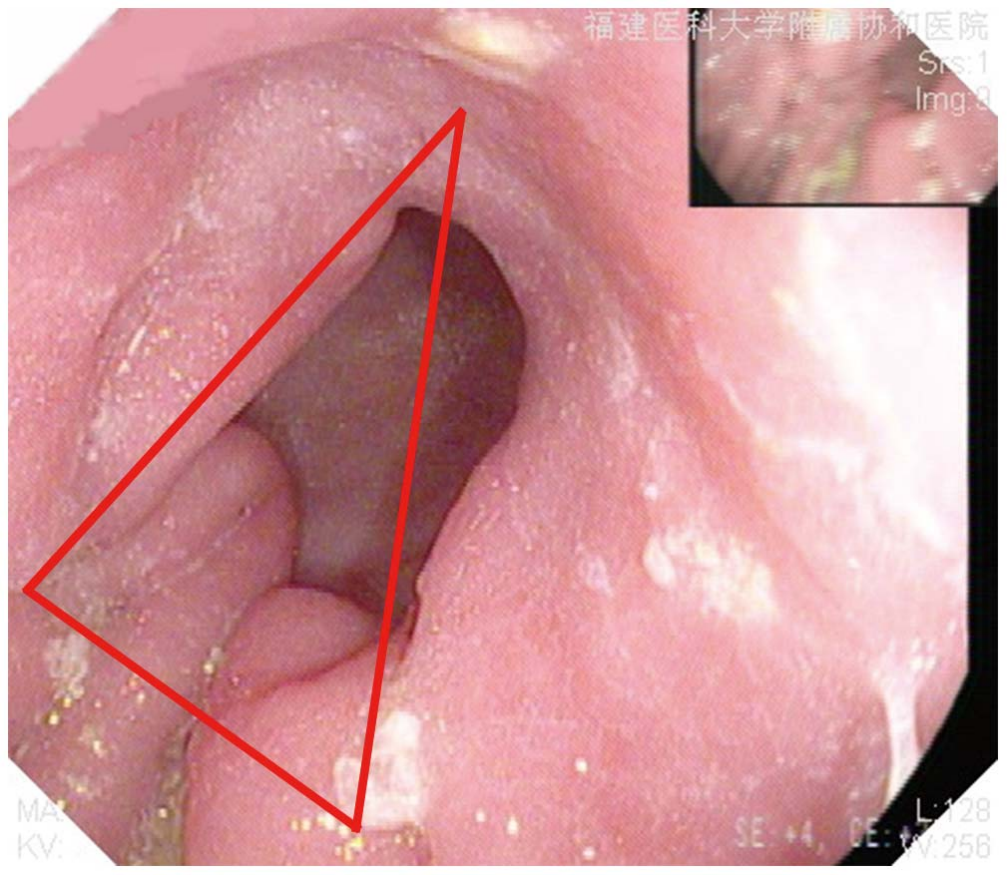
The gastroscope image of one patient underwent the modified delta-shaped gastroduodenostomy on postoperative 3 months.

A retrospective analysis was performed, using a prospectively maintained comprehensive database to collect the clinicopathological and follow-up data for all patients. Clinical and pathological staging were in accordance with the American Joint Committee on Cancer (AJCC) seventh edition of Gastric Cancer tumor, node, metastasis (TNM) Staging. [7] The anastomosis was checked for leakage on postoperative day 7–9 by performing an upper gastrointestinal radiograph with diatrizoate meglumine as the contrast medium. The anastomosis size was defined as the inner diameter of the anastomosis, measured on upper gastrointestinal radiography films in which the anastomotic site was fully filled with contrast medium. (Fig. 4) Anastomoses were evaluated after three months by gastroscopy. (Fig. 5) Postoperative follow-up was performed every 3 months for 2 years. Most patient routine follow-ups consisted of physical examination, laboratory tests (including CA19-9, CA72-4, and CEA levels), chest radiography, abdominopelvic ultrasonography or CT, and an annual endoscopic examination. All patients were observed until death or the last follow-up date of February 28, 2014.

### Ethics Statement

Ethics committee of Fujian medical union hospital approved this retrospective study. Written consent was given by the patients for their information to be stored in the hospital database and used for research.

### Statistical analysis

All of the statistical analyses were performed using the Statistical Package for the Social Sciences (SPSS), version 18.0 for Windows (SPSS Inc, Chicago, USA). Data are expressed as the means ± standard deviations. Categorical variables were analyzed by the Chi-square test, while continuous variables were analyzed by Student’s *t*-test. To evaluate predictive factors for postoperative morbidity, multivariate analysis was performed using binary logistic multiple regression tests using dummy variables. P values <0.05 were considered statistically significant.

## Results

### Clinicopathological characteristics of patients

The eligible patients included 74 males and 28 females, with a mean age 60.0±12.0 years (range 32 to 84 years) and a mean body mass index (BMI) 22.2±3.2 kg/m^2^ (range 16.2 to 31.6 kg/m^2^). The preoperative hemoglobin average of all patients was 126.5±22.4 g/L (range 58.0 to 168.0 g/L). The white blood cell count was 6.1±1.9*10∧9/L (range 1.5 to 13.1*10∧9/L), and the albumin was 41.0±4.6 g/L (range 27.9 to 50.4 g/L). Preoperative nutritional support was performed on 34 patients, while preoperative blood products transfusion including human blood albumin or suspended red blood cells was performed on 5 patients. Eleven patients were diagnosed as GC with hemorrhage (presented with melena) and 1 with pyloric obstruction. Thirty-nine patients with comorbidity included 22 with cardiovascular diseases (including high blood pressure, coronary heart disease and arrhythmia), 4 with diabetes, 1 with pulmonary diseases (including chronic obstructive pulmonary disease (COPD) and bronchiectasis), 1 with gout, 1 with hypothyroidism, 1 with cardiovascular diseases coexisting with diabetes and gout, 6 with cardiovascular diseases and diabetes, 1 with diabetes and hyperthyroidism, 1 with diabetes and hyperthyroidism, and 1 with cardiovascular and pulmonary diseases. All of the patients underwent histologically complete (R0) resections. The mean tumor size was 35.5±20.2 mm. The pathological TNM stages included IA (n = 39), IB (n = 21), IIA (n = 15), IIB (n = 11), IIIA (n = 9) and IIIB (n = 7). The pathological differentiation included high differentiation (n = 10), middle differentiation (n = 35), low differentiation (n = 36) and undifferentiation (n = 18). (Table 1).

**Table 1:** Clinicopathological Characteristics of patients undergoing TLDG with modified DSG for GC (n (%)).

Demographics	n = 102	Oncologic outcomes	n = 102
Age(years)	60.0±12.0	Tumor size(mm)	35.5±20.2
Gender		AJCC pT stage	
Male	74(72.6)	T1	46(45.1)
Female	28(27.5)	T2	28(27.5)
BMI(kg/m2)	22.2±3.2	T3	24(23.5)
Hemoglobin(g/L)	126.5±22.4	T4a	4(3.9)
Albumin(g/L)	41.0±4.6	AJCC pN stage	
White blood cell count(10∧9/L)	6.1±1.9	N0	59(57.8)
Preoperative nutritional support	34(33.3)	N1	24(23.5)
Preoperative BPT	5(4.9)	N2	15(14.7)
With hemorrhage	11(10.8)	N3	4(3.9)
With pyloric obstruction	1(1.0)	AJCC pTNM stage	
History of abdominal operation	18(17.7)	IA	39(38.2)
Comorbidity disease	39(38.2)	IB	21(20.6)
Cardiovascular	31(30.4)	IIA	15(14.7)
Diabetes	12(11.8)	IIB	11(10.8)
Pulmonary	3(2.9)	IIIA	9(8.8)
Gout	2(2.0)	IIIB	7(6.9)
Hypothyroidism	1(1.0)	Pathology	
Hyperthyroidism	1(1.0)	Differentiation	82(80.4)
–	–	Undifferentiation	20(19.6)

TLDG, totally laparoscopic distal gastrectomy; DSG, delta-shaped gastroduodenostomy; GC gastric cancer; BMI, body mass index; BPT, blood products transfusion; AJCC, American Joint Committee on Cancer 7th; pTNM, pathological tumor node metastasis.

### Surgical outcomes

Modified DSG in TLDG for GC was successfully completed in all patients, with none of these patients requiring conversion to open surgery. D1+ LN dissection was performed on 9 patients, while D2 was performed on 93 patients. The number of dissected lymph nodes per patient was 35.1±11.1. The mean operation time was 150.6±30.2 min, the mean anastomosis time was 12.2±4.2 min, the mean blood loss was 48.2±33.2 ml, and the mean times to first flatus, fluid diet, soft diet and postoperative hospital stay were 3.8±1.3 days, 5.0±1.0 days, 7.4±2.1 days and 12.0±6.5 days, respectively. The average size of the anastomosis was 30.4±3.6 mm. (Table 2).

**Table 2:** Surgical outcomes of patients undergoing TLDG with modified DSG for GC.

Items	n = 102(%)
Conversion to open surgery	0 (0.00)
Extent of LN dissection(n, D1+/D2)	9/93
Number of dissected LNs (per case)	35.1±11.1
Operation time(min)	150.6±30.2
Anastomosis time(min)	12.2±4.2
Intraoperative blood loss(ml)	48.2±33.2
Time to first flatus (days)	3.8±1.3
Resume liquid diet time (days)	5.0±1.0
Resume soft diet time (days)	7.4±2.1
Postoperative hospital stay (days)	12.0±6.5
Anastomosis size (mm)	30.4±3.6

LN, lymph node. DSG, delta-shaped gastroduodenostomy. TLDG, totally laparoscopic distal gastrectomy. GC gastric cancer.

### Postoperative complications and the predictable risk factors of that

Two patients experienced a minor anastomotic leakage after surgery; no patient experienced any complications around the anastomosis such as anastomotic stricture and anastomotic hemorrhage. The rate of anastomosis-related complications was 1.96%. The 2 patients with leakage presented with the symptoms of preoperative hemorrhage, such as melena, with histologically proven T3N2 undifferentiated and T4aN2 low differentiated gastric adenocarcinoma, respectively. Both leakages occurred at the gastric greater curvature site of the common closed edge on 9 days after gastrectomy and were cured after conservative treatment for 36 days and 28 days, respectively. Other complications occurred in 6 cases including 1 inflammatory intestinal obstruction with respiratory infection, 1 lower incomplete gastrointestinal obstruction, 1 gastric atony, 1 gastric atony with abdominal infection, and 2 respiratory infections. The overall morbidity rate was 7.84%. (Table 3) All of these postoperative complications were successfully treated by conservative methods. There were no deaths in all of the patients. Univariate analysis showed that age, gastric cancer with hemorrhage and cardiovascular disease combined were significant factors that affected postoperative morbidity (P<0.05). Multivariate analysis found that gastric cancer with hemorrhage was the independent risk factor for the postoperative morbidity (P = 0.042). (Table 4).

**Table 3:** Postoperative complications of patients undergoing TLDG with modified DSG for GC.

Items	n = 102
**Anastomosis-related complications (n)**	
Anastomotic leakage	2
Anastomotic stricture	0
Anastomotic hemorrhage	0
**Other complications (n)**	
Postoperative celiac infection	1
Respiratory infection	3
Inflammatory intestinal obstruction	1
Lower incomplete gastrointestinal obstruction	1
Gastric atony	2
Overall postoperative complications*[n (%)]	8(7.84%)
Postoperative mortality [n (%)]	0(0%)

TLDG totally laparoscopic distal gastrectomy; DSG delta-shaped gastroduodenostomy; GC gastric cancer.

* Repeated cases were not included.

**Table 4:** Risk factors influencing complications after TLDG with modified DSG for GC.

Items	With complications (n = 8)	Without complications (n = 94)	Univariate analysis p	Multivariate analysis	
				95% CI	p
Gender			0.162		
Male	8(100%)	66(70.2%)			
Female	0(0%)	28(29.8%)			
Age(years)	69.1±9.9	59.2±11.9	0.024	0.953–1.138	0.368
BMI	28.80±2.99	22.29±3.19	0.234		
Hemoglobin (g/L)	128.13±17.89	126.56±23.21	0.853		
White blood cell coun (10∧9/L)	7.24±2.87	6.04±1.86	0.099		
Albumin (g/L)	41.01±3.24	40.89±4.72	0.941		
With hemorrhage			0.002	0.027–0.939	0.042
Yes	4(50%)	7(7.4%)			
No	4(50%)	87(92.6%)			
Preoperative NS			0.896		
Yes	2(25%)	32(34%)			
No	6(75%)	62(66%)			
Preoperative BPT			0.854		
Yes	1(12.5%)	4(4.3%)			
No	7(87.5%)	90(95.7%)			
History of abdominal operation			0.378		
Yes	0(0%)	18(19.1%)			
No	8(100%)	76(80.9%)			
Comorbidity disease			0.064		
Yes	6(75%)	33(35.1%)			
No	2(25%)	61(64.9%)			
Cardiovascular			0.014	0.041–2.847	0.322
Yes	6(75%)	25(26.6%)			
No	2(25%)	69(73.4)			
Diabetes			1.000		
Yes	1(12.5%)	11(11.7%)			
No	7(87.5%)	83(88.3%)			
Pulmonary			0.564		
Yes	1(12.5%)	2(2.1%)			
No	7(87.5%)	92(97.9%)			
Operative time (min)	139.4±22.4	151.5±30.7	0.278		
Anastomosis time(min)	11.9±4.5	12.2±4.2	0.822		
Number of dissected LNs (per case)	35.9±9.3	35.0±11.3	0.835		
Intraoperative blood loss (mL)	45.0±31.2	48.6±33.0	0.769		
Tumor size(mm)	40.0±22.0	35.1±20.1	0.516		
Pathology			0.993		
Differentiated	6(75%)	77(81.9%)			
Undifferentiated	2(25%)	17(18.1%)			
pT			0.445		
T1	2(25%)	43(45.1%)			
T2–T4a	6(75%)	51(54.3%)			
pN			1.000		
N0	5(62.5%)	53(56.4%)			
N1–N3	3(37.5%)	41(43.6%)			
pTNM			0.924		
I	4(50%)	55(58.5%)			
II–III	4(50%)	39(41.5%)			

TLDG totally laparoscopic distal gastrectomy; DSG delta-shaped gastroduodenostomy; GC gastric cancer; CI, confidence interval; NS, nutritional support; BPT, blood products transfusion; LN, lymph node.

### Late follow-up

All of the patients (100%) were followed-up for 1 to 13 months. At a median follow-up of 7 months, no patient had died or experienced recurrent or metastatic disease. Moreover, there was no abdominal distension, nausea, vomiting, acid reflux or other symptoms in all patients. All anastomoses were patent in the gastroscopy and no complications such as anastomotic stricture occurred. Bile reflux into the remnant stomach was observed endoscopically in 73.5% of our patients (21/102), but the pathological changes of alkaline reflux gastritis didn’t occur in the gastroscopy and the corresponding symptoms were not observed in these patients.

## Discussion

Surgical resection is the primary treatment method for GC. Operation methods that reduce surgical trauma and optimize patient quality of life are preferred. Since the introduction of laparoscopy-assisted distal gastrectomy (LADG) for GC in 1994, [1] laparoscopic surgery has become widely used in patients with GC, with satisfactory surgical outcomes. [8]–[14] B-I anastomosis is preferred for reconstruction after LADG because of its relative simplicity and physiological advantages, which include allowing food to pass through the duodenum and reducing the postoperative incidence of cholecystitis and cholelithiasis. [15]–[17] However, anastomosis during totally laparoscopic surgery is technically difficult. DSG, a method of B-I anastomosis after TLDG using only endoscopic linear staplers, [2] has been utilized in several Asian countries, including Japan and Korea, [3]–[5] due to its relative simplicity and satisfactory results. [18] TLDG with DSG has been shown to be more minimally invasive than LADG, [19]–[21] especially in obese patients. [5], [20], [22].

However, because the higher technical demand is needed and most surgeons still doubt for its safety, this method has not been carried out extensively at present. The laparoscopic suturing used in conventional DSG to produce an involution of the common stab incision was found to require a relatively long period of time. In addition, the surgeons must be proficient at the suturing techniques under laparoscopy, or it may affect the result of the anastomosis due to unfavorable suturing and may increase the surgical trauma. Therefore, we proposed the modified technique to omit this step. Instead, the procedure requires only the instruments of the surgeon and the assistant to directly grasp the tissue and efficiently accomplish the involution of the common stab incision. Thus, it simplifies the operation procedures, shortens anastomosis times and reduces surgical trauma. Moreover, previous studies showed that the rates of anastomotic leakage after DSG ranged from 0.42% to 8.5%. [3], [18], [20]–[23] There were also anastomotic bleeding and anastomotic stricture reported in the literature. [20]–[23] The postoperative morbidity rates of anastomosis-related complications ranged from 1.0% to 12.7%. [3], [18], [20]–[23] Considering the conventional method resulted in a duodenal blind side and two intersections of the cutting edge of the remnant stomach with the duodenum and the common closed edge, which may cause poor blood supply to the duodenal stump and yield two weak points of the anastomosis and may increase the risk of anastomosis-related complications such as anastomotic leakage and anastomotic bleeding, the conventional DSG procedure was modified based on our surgical experience. In the modified method, the duodenal cutting edge was completely resected to avoid the poor blood supply to the duodenal stump, and the appearance of the anastomoses was also changed from two intersections to only one as an inverted T-shape, which could decrease the anastomotic weak point and produce a more stable structure. Based on the above, we considered that the modified DSG would reduce the risk of anastomosis-related complications. Our results also showed that no patients of the 46 cases of early GC experienced any anastomosis-related complications such as anastomotic leakage and anastomotic bleeding. Of all the patients, only 2 elderly patients (1.96%) with late-stage GC experienced a minor anastomotic leakage 9 days after surgery and were managed conservatively. No other complications around the anastomosis, such as anastomotic hemorrhage, occurred. With the modified technique, as long as the cutting line of the closure of the common stab incision is maintained perpendicular to the cutting edge of the remnant stomach, the anastomosis size would not be decreased and anastomotic stricture would not develop. The average size of the anastomosis was 30.4±3.6 mm in our study and no anastomotic stricture occurred in the late follow-up. Although the incidence of bile reflux was 20.6%, no patient had corresponding symptoms and remnant gastritis. Thus, while bile reflux was observed in some patients, it did not appear to be a clinically critical problem in our study.

The simplified operation procedures can obtain a simpler process, and the reduced potential safety risk can increase the safety of operation. The early clinical results indicated that the modified DSG procedure was safe and feasible in TLDG for GC, with a mean operation time, mean blood loss and postoperative morbidity that were comparable with other reports. [3]–[5], [18], [20] The average time of anastomosis was 12.2±4.2 min, and the surgical outcomes were acceptable. Several risk factors were identified for the postoperative complications. The older patients were often under poor nutritional status with substantial organ diseases such as diseases involving the heart, lung, brain and kidney. Furthermore, GC with hemorrhage may further worsen the clinical condition to produce a poorer nutritional status in patients. Thus, the risk of surgery and the rates of the postoperative complications were increased under those conditions. The patients with leakage in our study both presented with the symptoms of preoperative hemorrhage such as melena and the leakages both occurred at the gastric greater curvature site of the common closed edge, which was shown to have a relatively high rate of anastomotic leakage in previous research. [23] This suggested that during the surgery, careful attention should be paid to the blood supply on the gastric greater curvature site of the anastomoses, especially for the patients with preoperative hemorrhage in GC, and the leakage could be prevented by the placement of an intraoperative reinforcing suture and perioperative active management.

In conclusion, the modified DSG was technically safe and feasible in patients with GC undergoing TLDG. The procedure decreased the anastomotic weak points and avoided the poor blood supply to the duodenal stump; it may be promising in these patients and is easier to perform with acceptable surgical outcomes. Longer follow-up is needed in the future to confirm these results. To be accepted as a first-line treatment in TLDG for GC, well-designed prospective randomized controlled trials comparing short-term and long-term outcomes in a larger number of patients are necessary.
